# Case Report – Successful Thrombectomy After Critical Resuscitation Following a Cardiac Arrest

**DOI:** 10.1161/SVIN.122.000407

**Published:** 2022-07-18

**Authors:** Rohan Mathur, Olanrewaju Agbe‐Davies, Victor Urrutia, Justin M. Caplan, Juan Ricardo Carhuapoma

**Affiliations:** ^1^ Division of Neurosciences Critical Care Departments of Anesthesiology & Critical Care Medicine and Neurology The Johns Hopkins University School of Medicine Baltimore MD; ^2^ Department of Neurology Northeast Georgia Medical Center Gainesville GA; ^3^ Division of Vascular Neurology Department of Neurology The Johns Hopkins University School of Medicine Baltimore MD; ^4^ Department of Neurosurgery The Johns Hopkins University School of Medicine Baltimore MD

## Abstract

Sparse evidence exists to support a delayed attempt at thrombectomy after a periprocedural cardiac arrest or acute medical decompensation. This case highlights the presentation of a 23‐year‐old woman with dilated cardiomyopathy, who initially presented with a left middle cerebral artery stroke syndrome, but who had a cardiac arrest in the angiogram suite prior to the procedure starting. After aggressive resuscitation in the critical care unit, repeat imaging showed a persistent perfusion deficit in the left middle cerebral artery territory and she successfully underwent a thrombectomy. The case highlights that critical care efforts, even after a severe initial presentation, and significant resuscitative efforts in the intensive care unit setting, can still lead to a successful thrombectomy, and that eligibility for thrombectomy can be reassessed as the patient stabilizes.

A 23‐year‐old woman, with history of dilated cardiomyopathy due to a preceding viral carditis, with biventricular heart failure and a left ventricular ejection fraction of 15%, not on anticoagulation, and chronic kidney disease, presented with acute onset of global aphasia and right hemiplegia (National Institutes of Health Stroke Scale 18). She was last known well at her intact neurological baseline 1 hour prior to arrival, and her head CT without contrast had an Alberta Stroke Program Early CT score of 8. She had thrombocytopenia with a platelet count of 96 000 mm^−3^ thus iv tPA was assessed to be contraindicated and not administered. Her initial labs were also notable for serum sodium of 123 mEq/L. CT‐angiogram of her head and neck demonstrated occlusion of the main arterial trunk of the left middle cerebral artery. She was transferred emergently to our comprehensive stroke center. On arrival to the interventional neuroradiology suite, she was intubated. Prior to groin puncture, she developed a sudden onset ventricular tachycardia followed by cardiac arrest. Cardioplmonary resuscitation was promptly started and return of spontaneous circulation was achieved in 4 minutes. No further attempt at thrombectomy was made at that time, as she was deemed too hemodynamically unstable and she was brought to the intensive care unit for further stabilization.[Fig svi212344-fig-0001]

While in the Neurosciences Critical Care Unit, she was started on vasopressors for cardiogenic shock. Her urine output declined, and her bloodwork showed multiple electrolyte derangements and acidemia secondary to lactic acidosis. She was started on continuous renal replacement therapy. Over a period of approximately 16 hours, her electrolytes normalized, her acidemia resolved, and her vasopressor requirement resolved. At this time, 22 hours from last known well, her neurological exam remained consistent with a left middle cerebral artery syndrome. She was taken for a hyperacute magnetic resonance imaging brain and MR‐perfusion, that showed a target‐apparent diffusion coefficient core of 45 mL involving the left basal ganglia, but arterial spin labeling perfusion maps demonstrated an ischemic penumbra encompassing the entire left middle cerebral artery distribution (Figure, panels A and B). Given the persistent perfusion deficit and mismatch, along with correction of the acute metabolic abnormalities, she met DAWN criteria to pursue attempted intraarterial thrombolysis.^1^ Further discussions were emergently held with her husband, who was making decisions on her behalf due to aphasia. With his consent, she was taken for a cerebral angiogram, and had a successful thrombectomy with thrombolysis in cerebral infarction‐2b reperfusion (Figure, panels C and D). Within a few hours postprocedure, she was extubated. Her neurological exam postthrombectomy improved significantly with intact comprehension, mostly comprehensible speech with occasional word finding difficulty and some residual mild right‐sided weakness. She was eventually discharged home from the hospital. Five months later, in the setting of further decompensated heart failure, she and her husband both participated in extensive palliative care discussions, and per her wishes, she was transitioned to comfort care, and died in an inpatient hospice setting.

**Figure svi212344-fig-0001:**
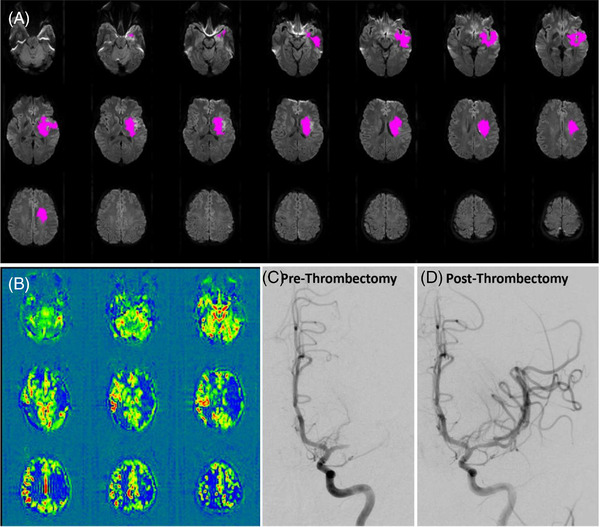
**Relevant neuro‐imaging showcasing key points in the case**. **A**, MRI brain with perfusion imaging, performed after aggressive resuscitation in the ICU, demonstrated an approximately 45cc core infarct (pink). **B**, The corresponding ASL perfusion map demonstrated a perfusion deficit in the left MCA territory that was significantly larger than the identified core. **C**, Cerebral angiogram prethrombectomy, (**D**) cerebral angiogram postthrombectomy of a left M1 thrombus with TICI‐2b reperfusion. ASL indicates arterial spin labeling; ICU, intensive care unit; MCA, middle cerebral artery; MRI, magnetic resonance imaging; and TICI, thrombolysis in cerebral infarction.

## DISCUSSION

Sparse evidence exists to support a delayed attempt at thrombectomy after a periprocedural cardiac arrest or acute medical decompensation. The case highlights that critical care efforts, even after a severe initial presentation, and significant resuscitative efforts in the intensive care unit setting, can still lead to a successful thrombectomy, and that eligibility for thrombectomy can be reassessed as the patient stabilizes.

This case also highlights the role thrombectomy has in patients with severe underlying comorbidities. The ethical principles of providing benefit and avoiding harm support the idea of offering thrombectomy in situations when the patient's underlying comorbidity may be incurable or progressive. A similar case discussing palliative thrombectomy in a patient with a left middle cerebral artery syndrome and acute large vessel occlusion with an underlying history incurable nonsmall cell lung cancer, also supported the decision to pursue thrombectomy based on a similar ethical decision‐making framework.^2^ In making the decision to proceed with another attempt at thrombectomy, we came to the consensus that the decision to perform thrombectomy was ethically justifiable based on conversations with her husband about her goals of care, and with the appreciation that profound aphasia and right sided weakness would significantly impact her quality of life. On follow‐up, her husband expressed gratitude for the few extra months of meaningful time communicating with his wife. As a result of this intervention, she not only gained mobility and the means to communicate, she was also able to notably participate in her own medical and goals of care discussions, thereby preserving her decision‐making autonomy.

## Sources of Funding

None.

## Disclosure

None.
